# Emotion Regulation Among Adolescents With Pediatric Depression As a Function of Anxiety Comorbidity

**DOI:** 10.3389/fpsyt.2019.00722

**Published:** 2019-10-07

**Authors:** Roberta Dochnal, Ágnes Vetró, Enikö Kiss, Ildikó Baji, Eszter Lefkovics, Lauren M. Bylsma, Ilya Yaroslavsky, Jonathan Rottenberg, Maria Kovacs, Krisztina Kapornai

**Affiliations:** ^1^Department of Child and Adolescent Psychiatry, Pediatrics and Child Health Center, University of Szeged, Szeged, Hungary; ^2^Department of Psychiatry, School of Medicine, University of Pittsburgh, Pittsburgh, PA, United States; ^3^Department of Psychology, Cleveland State University, Cleveland, OH, United States; ^4^Department of Psychology, University of South Florida, Tampa, FL, United States

**Keywords:** pediatric depression, anxiety depression comorbidity, emotion regulation, internalizing psychopathology, adolescent

## Abstract

**Background:** Both depression and anxiety (two of the most common internalizing psychopathologies among youths) are associated with difficulties in emotion regulation (ER). Little is known about whether anxiety as a comorbid condition has an effect on the habitual use of different ER strategies in youngsters with depression histories. We aimed 1) to compare ER in adolescents with histories of childhood onset major depressive disorder (MDD) with and without comorbid anxiety and 2) to examine whether certain ER response clusters (Cognitive, Social, and Behavioral/Physical) characterize comorbid children and adolescents.

**Methods:** We analyzed data on 217 youth (11–18 years old) with depression history: 85 subjects with lifetime anxiety comorbidity (comorbid group) and 132 without lifetime anxiety (non-comorbid group). Psychiatric diagnosis was established by a comprehensive *Diagnostic and Statistical Manual of Mental Disorders* (DSM) IV-based diagnostic procedure. ER strategies were examined *via* the self-rated “Feelings and Me” Child version questionnaire (FAM-C).

**Results:** The comorbid group used maladaptive ER strategies significantly more frequently than the non-comorbid youngsters. The Behavioral/Physical and Social ER skills, especially those reflecting social withdrawal and self-harm, were responsible for the higher maladaptive scores.

**Limitations:** Because our study is a cross-sectional analysis, we have no information about the development or the onset of maladaptive ER strategies. Therefore, we were unable to examine whether maladaptive ER was a risk factor or a consequence of the internalizing psychopathology and comorbidity.

**Conclusions:** Comorbid anxiety worsens the impaired use of ER strategies in depression-prone youths. Further longitudinal research is needed to explore the causal role of dysfunctional ER in the development of internalizing psychopathology.

## Introduction

The ability to regulate emotions and attenuate negative emotions is considered fundamental to healthy child development and functioning (1, 2). The concept and development of emotion regulation (ER) have been extensively discussed by several authors (1–6). According to Cole et al. (4), ER consists of several cognitive, behavioral, and social self-regulatory responses that can change the activated emotion by modulating its valence, intensity, or time course. Appropriate adaptive ER strategies (e.g., distraction, cognitive reappraisal, and seeking interpersonal support) attenuate dysphoria and facilitate functioning (7–9), while maladaptive responses (e.g., rumination and suppression) prolong and exacerbate dysphoria (8, 10). Furthermore, it is established in the literature that poor ER is implicated in most forms of childhood psychopathology; a variety of ER deficits are present in samples of children with internalizing disorders (11).

Depressed youngsters use a greater number of maladaptive mood repair strategies and fewer adaptive ones than do healthy controls (12, 13). The study by Kovacs et al. (8), based on clinical samples, indicates that maladaptive skills are correlated with a worsening of depression symptoms and increase the probability of recurrent depressive episodes. ER difficulties can persist even after depression has remitted. Our research group previously showed that both remitted and currently depressed young adult probands reported a greater number of maladaptive ER responses to sadness than did controls (8). ER difficulties have also been reported in children and adolescents with histories of depression than in healthy controls (13). In addition, younger depressed children (kindergarten to eighth grade) have been found to use maladaptive responses to regulate emotions more frequently than non-depressed children (14).

ER also seems to be impaired in individuals with anxiety symptoms (11, 15). A growing body of literature suggests that anxiety, similar to depression, is associated with the decreased use of adaptive regulation responses and increased use of maladaptive responses, in both youth and adults (e.g., 16–19). For example, Suveg and Zeman (20) found that children with anxiety disorders were less successful in controlling negative emotions than were healthy controls. Anxious children have lower levels of emotional understanding and more difficulty in regulating worry, sadness, and anger (2). According to Carthy et al. (21), children with anxiety disorder have greater negative emotionality and more deficits in using reappraisal in negative emotional situations. Mennin and colleagues (22) found in adult population sample that patients (mean age 19.52 years) with generalized anxiety disorder (GAD) had low self-soothing ability following a negative emotional experience.

Depression and anxiety disorders are the most common comorbid mental disorders. Based on comprehensive epidemiological data, the estimated rate of comorbid anxiety disorders in children and adolescents with depression ranges from 30% to 75% (23, 24). Comorbidity estimates in clinical samples can be as high as 86% (25–27).

Research on the impact of comorbidity of depression and anxiety disorder suggests that patients with comorbidity have greater impairment and symptom severity, more chronic course of illness, and decreased response to treatment relative to patients with these conditions in isolation (28–31). Furthermore, youths with both anxiety and depressive disorders had high risk for suicide attempt (32).

Previous ER research on anxiety and depression comorbid patients focused mainly on maladaptive cognitive skills and the role of ruminative thinking (33, 34). Results showed that patients with comorbidity use maladaptive ER strategies, such as rumination, suppression, and avoidance, more often than do adaptive ones (11, 12, 15, 35). Garnefski and Kraaij (37) investigated an adolescent community sample. Adolescents with symptoms of depression and anxiety used rumination, catastrophizing, other-blame, and self-blame, whereas adolescents with only depression used self-blame and rumination. Adaptive ER strategies (positive reappraisal and refocusing on planning) were inversely related to both depressive and anxiety symptoms, while positive refocusing was inversely related only to depressive symptoms. Garnefsky et al. (38) previously showed that the above maladaptive coping strategies were strongly related to symptoms of depression and anxiety in both adolescents and adults. A study conducted by d’Avanzato and Joormann, (39) in adult population with social anxiety and depression found that a higher level of rumination and a lower level of reappraisal were specific for patients with major depressive disorder (MDD).

The relationship between self-blame and depressive and anxiety symptoms was stronger in adolescents than in adults. Positive reappraisal, an adaptive coping strategy, was less often used by adolescents, indicating that they were less likely than adults to try to add positive meaning to a negative life event (38).

In summary, anxiety and depressive disorders are both associated with abnormalities in the processing and regulation of emotions. Understanding the link between these dysfunctional strategies and the psychopathology of children and adolescents with depression and anxiety may facilitate the development of more efficient prevention and treatment approaches.

The goal of this study was to compare the ER strategies of youngsters with histories of MDD with and without comorbid anxiety disorders. Specifically, we aimed 1) to compare the adaptive and maladaptive ER repertoires of youngsters with histories of MDD with and without comorbid anxiety and 2) to examine whether certain ER response clusters (Cognitive, Social, and Behavioral/Physical) characterize comorbid children and adolescents.

## Materials and Methods

This study was approved by the institutional review boards of the University of Pittsburgh, USA, and the University of Szeged, Hungary. Parents provided written informed consent, and youths provided either assent or consent (depending on their ages) before any data were gathered. All procedures, schedules, rating scales, and instruments used in this study were first developed in English, translated to Hungarian, and then retranslated to English by bi-lingual child psychiatrists and clinical psychologists. Original and back-translated versions were compared, and discrepancies were resolved.

### Sample

The subjects in our study were selected from a genetic and other risk factor study of childhood onset depression (Program Project Hungarian study) described elsewhere (40). The original study entry criteria were as follows: *Diagnostic and Statistical Manual of Mental Disorders* (DSM) IV MDD, 7 to 14 years old at initial assessment, no evidence of mental retardation or major systemic medical disorder, availability of at least one biological parent, and having at least one sibling aged 7–18 years.

Children in the cohort were clinical patients at 23 child psychiatry inpatient and outpatient facilities in Hungary at the time of study entry. For the present paper, we analyzed data on 217 children who were invited to participate in the Biobehavioral Inflexibility and Risk for Juvenile-onset Depression study. Their age ranged from 11 to 18 years at the time of assessment.

### Measurement

#### Diagnostic Procedures

The Interview Schedule for Children and Adolescents—Diagnostic Version (ISCA-D) was used for establishing psychiatric diagnoses. ISCA-D is an extension and modification of the Interview Schedule for Children and Adolescents (ISCA) (41). It is a semi-structured interview assessing lifetime psychiatric disorders and current psychiatric status along with the onset and offset dates of each disorder in youths on the basis of DSM-IV (42). Psychiatric diagnoses were evaluated over the subject’s lifetime. The intake interviews were assessed during the original Program Project study and covered the time frame from birth to the time of the interview. The 217 youths who participated in the present study were re-evaluated by the follow-up version of ISCA-D (FU-ISCA-D) to assess their current diagnoses and also their psychiatric histories since the previous interview. Therefore, diagnostic evaluation covers the time from birth to current assessment. All diagnoses, number of episodes, and age at first depressive disorder were also evaluated from birth till the timepoint of the actual assessment. Diagnostic evaluations were carried out by trained child psychiatrists and psychologists who completed 3 months of didactic and practical training in the ISCA-D semi-structured interview technique and rendered best-estimate psychiatric consensus diagnosis. As reported elsewhere, interviewers have achieved satisfactory inter-rater reliability (40, 43, 44).

#### Self-Rating Scales

Self-rating scales for the present study were administered at the time of Biobehavioral Inflexibility and Risk for Juvenile-onset Depression study, after the FU-ISCA-D interview, on the same day. Interviewers were available to help younger children as needed.

ER strategies were examined *via* the self-rated “Feelings and Me” Child (FAM-C) version questionnaire, which evaluates the use of responses to depressed, dysphoric mood (13, 44, 45). It is presumed that there is a trait-like characteristic style of the use of the ER response repertoires. Therefore, there is no timeframe for this questionnaire.

The FAM-C is suitable for ages 7–17 and lists a total of 54 depression-relevant mood repair strategies focusing on coping with sadness: 32 strategies that are “adaptive” (i.e., serve to downregulate/regulate sadness, e.g., listen to music) and 22 strategies that are “maladaptive” (i.e., serve to exacerbate sadness, e.g., hit myself). Besides the Total Adaptive and Maladaptive scores, the items reflect three regulatory domains—Behavioral/Physical (24 items), Social-Interpersonal (12 items), and Cognitive (18 items)—with adaptive and maladaptive strategies in each. Each response is scored 0, 1, or 2, corresponding to the choice of “not true,” “sometimes true,” or “often true.” We have previously reported good psychometric properties of this measure across clinical and non-clinical populations (13, 44).

In order to control for depressive symptoms, the Child Depression Inventory (CDI-R) was administered. CDI-R is a 27-item, self-rated, symptom-oriented scale suitable for youths aged 7 to 17, sensitive to changes in depressive symptoms over time, and is a useful index of the severity of the depressive episode. It measures depressive symptoms in the last 2 weeks (46).

### Statistical Analysis

We used SPSS Statistics 22.0 package for all the performed statistical analyses.

The percentage of missing data was very low: 0.4% in the whole dataset. By default, SPSS treated missing values as “missing,” and these items were not included in the statistical analyses.

To examine possible differences between groups on baseline characteristics, we used independent *t*-test for continuous and chi-square test for categorical variables. The general linear model (GLM)-univariate analysis was used to explore the differences in the ER responses between the groups. False discovery rate (Benjamini–Hochberg procedure) was used to control for multiple comparisons.

The *dependent variables* were Total Adaptive, Total Maladaptive, Adaptive Cognitive, Maladaptive Cognitive, Adaptive Social, Maladaptive Social, Adaptive Behavioral/Physical, and Maladaptive Behavioral/Physical scores as measured by the FAM-C.

*The independent variable was* group membership. The two groups were youth with any lifetime depression disorder and any lifetime anxiety disorder (comorbid group) and youth with any lifetime depression disorder and no lifetime anxiety disorder (non-comorbid group).

*Covariates* included CDI-R scores, number of MDD episodes, age, sex, and age of onset of first mood disorder (MDD or dysthymia). Based on the literature, depression severity and ER deficits are directly related (e.g., 8). Severity of depression can be reflected by CDI-R scores, number of MDD episodes, and age of onset of first mood disorder. We included these items as covariates, because these parameters were significantly different between the two groups and thus might have an influence on our hypothesis, namely, the relation between anxiety comorbidity and ER in depressed youths.

## Results

### Sample Description

**Figure 1:**
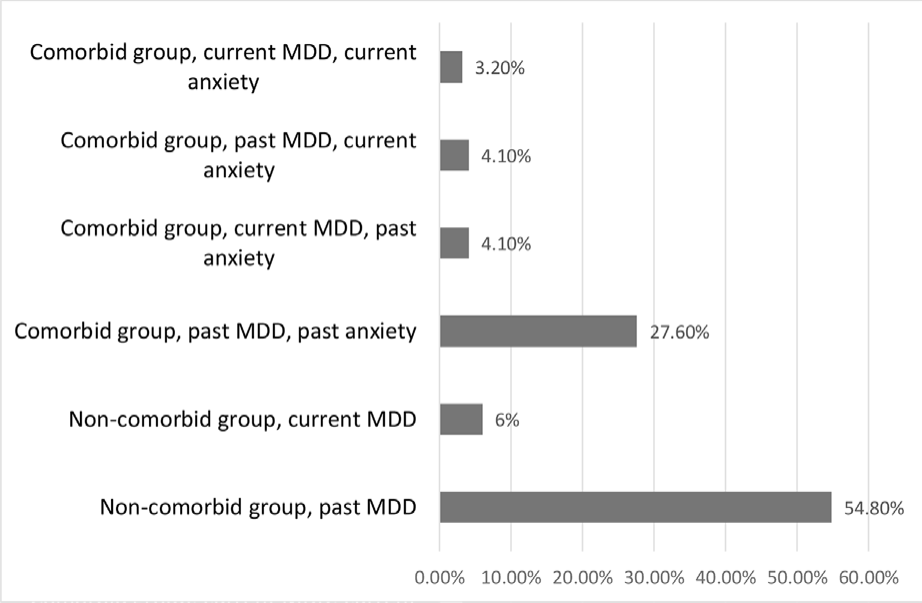
Comorbid vs. non-comorbid groups, compared based on current vs. past diagnoses of major depressive disorder (MDD) and anxiety disorder. MDD, major depressive disorder.

Our sample included 217 children (ages: 11–18 years; mean age: 17.01; SD: 1.39; gender distribution: 139 male and 78 female) who had had at least one lifetime MDD episode: 54.8% had one, 30% had two, and 15.2% had three or more MDD episodes. Of the whole sample, 13.4% were currently in depressive episode. The exact pattern of past and current MDD and anxiety episodes in our sample is shown in **Figure 1**. Patients with bipolar disorder (*n* = 6) were excluded from the statistical analysis.

The characteristics of the comorbid and non-comorbid groups are shown in **Table 1**. The most frequent anxiety disorders in the comorbid group were phobia (33 subjects, 38.82%), GAD (28 subjects, 32.94%), and separation anxiety disorder (25 subjects, 29.41%). Other comorbidities were anxiety not otherwise specified (NOS) (14 subjects, 16.47%), obsessive compulsive disorder (7 subjects, 8.23%), panic disorder (4 subjects, 4.7%), and post-traumatic stress disorder (4 subjects, 4.7%). Of the 85 comorbid patients, 50 subjects had one comorbid anxiety disorder, and 35 subjects had two or more comorbid anxiety disorders.

**Table 1:** Sample description non-comorbid vs. comorbid group.

	Non-comorbid group (*n* = 132) Mean (SD)	Comorbid group (*n* = 85) Mean (SD)	*p* value
Age (years)	17.04 (1.4)	16.97 (1.3)	0.73
Gender distribution	Male: 63.63%	Male: 64.7%	0.87
CDI-R total score	9.6 (6.3)	11.9 (8.2)	0.02*
Age of onset of first MDD episode (years)	9.21 (1.8)	8.75 (1.8)	0.08
Age of onset of first depressive disorder (MDD, dysthymia) (years)	9.05 (1.8)	8.4 (1.9)	0.03*
No. of MDD episodes	1.53 (0.7)	1.78 (0.8)	0.03*
No. of youth currently in MDD episode	13 (9.8%)	16 (18.8%)	0.06
Age of onset of earliest anxiety disorder (years)	–	8.08 (3.69)	

MDD, major depressive disorder; CDI-R, Child Depression Inventory.

*p ≤ 0.05.

The two groups did not differ in gender and age distribution, age of onset of first MDD episode, or the number of youths currently in MDD episode. However, the CDI-R scores, age of onset of first mood disorder, and the number of MDD episodes showed statistically significant differences in the two groups. Therefore, we used these variables as covariates in our group analyses (**Table 1**).

### Comparison of Total Adaptive and Maladaptive ER Scores in the Non-Comorbid vs. Comorbid Group

The Total Adaptive score of the FAM-C did not show statistically significant difference between the groups [*F*(1, 205) = 1.023, *p* = 0.313, partial *η*^2^ = 0.005], while the Total Maladaptive score was significantly higher in the comorbid compared with non-comorbid subjects [*F*(1, 205) = 5.269, *p* = 0.023, partial *η*^2^ = 0.025].

### Comparison of Subscales of Adaptive and Maladaptive ER Strategies in the Non-Comorbid vs. Comorbid Group

We found that none of the Adaptive subscales were significantly different between the groups: Cognitive [*F*(1, 204) = 0.648, *p* = 0.422, partial *η*^2^ = 0.03], Social [*F*(1, 204) = 0.287, *p* = 0.593, partial *η*^2^ = 0.001], and Behavioral/Physical [*F*(1, 202) = 0.699, *p* = 0.404, partial *η*^2^ = 0.003]. However, Maladaptive Social [*F*(1, 204) = 5.59, *p* = 0.019, partial *η*^2^ = 0.027] and Maladaptive Behavioral/Physical [*F*(1, 204) = 5.865, *p* = 0.016, partial *η*^2^ = 0.028] subscales were significantly higher in comorbid children (**Table 2**).

**Table 2:** FAM-C scores in the non-comorbid group vs. comorbid group

FAM-C scores	Group estimated mean (SE)		*p* value
	Non-comorbid	Comorbid	
Adaptive Scores Total (n = 212)	18.9 (0.76)	20.1 (0.94)	0.313
Cognitive (n = 211)	6.4 (0.31)	6.8 (0.38)	0.422
Social (n = 212)	3.3 (0.23)	3.5 (0.28)	0.593
Behavioral/Physical (n = 210)	9.2 (0.41)	9.7 (0.51)	0.404
Maladaptive Scores Total (n = 212)	10 (0.5)	11.9 (0.62)	0.023*
Cognitive (n = 210)	3.9 (0.27)	4.4 (0.33)	0.306
Social (n = 212)	2.5 (0.17)	3.2 (0.21)	0.019*
Behavioral/Physical (n = 212)	3.5 (0.22)	4.4 (0.27)	0.016*

Total, Social, Behavioral/Somatic, adaptive, and maladaptive ER scores (FAM-C).

FAM-C, feelings and me questionnaire, child version.

p values less than 0.0375 were considered significant after false discovery rate (Benjamini–Hochberg procedure) adjustment.

n = sample size analyzed (according to missing values by SPSS correction).

*statistically significant.

When we looked at specific items of the above-mentioned maladaptive subscales, the item reflecting social withdrawal in the Social subscale and the item reflecting self-harm in the Behavioral/Physical subscale were more frequently used by comorbid youth (data not shown). The Maladaptive Cognitive subscale was not significantly different across groups. However, one of the cognitive items similar to rumination was significantly more frequently endorsed by the patients in the comorbid group (think of being sad) [*F*(1, 205) = 4.794, *p* = 0.03, partial *η*^2^ = 0.023].

## Discussion

The aim of our study was to examine whether anxiety as a comorbid condition was associated with the use of different ER strategies in youngsters with histories of depression. Our study included a large clinical sample of Hungarian children and adolescents with depressive history, of whom about 39% had lifetime comorbid anxiety disorder as well.

Poor regulation of emotions appears to be a factor common to anxiety and depression, but the nature of ER in comorbid patients has not been fully characterized. In fact, there is scant information about how anxiety as a comorbid condition affects the habitual use of different ER strategies in depressed youngsters. Queen and Ehrenreich-May (47) found that adolescents with anxiety and depression had poorer emotional awareness, greater emotional suppression, greater reluctance to express negative emotions, and greater inhibition of sadness than are patients with anxiety disorder. Research by Burklund et al. (48) in adults revealed altered ER in patients with comorbid social phobia and depression. Aldao et al. (12) concluded, also in adults, that maladaptive ER strategies (e.g., rumination and avoidance) had strong associations with psychopathology including depression and anxiety.

The current study extended previous findings in several ways: We examined multiple adaptive and maladaptive ER strategies; we used a large clinical sample of youngsters with childhood onset depression; and our sample was carefully diagnosed by trained psychiatrists and psychologists. We found that anxiety comorbidity in youngsters with lifetime depression was associated with dysfunctional ER, as children with comorbidity used maladaptive ER responses more frequently than non-comorbid peers. Our results are in line with findings from previous studies that showed more frequent use of maladaptive ER strategies in youths with comorbid depression and anxiety symptoms (11, 15, 36, 37, 47). It has been reported that the treatment of depression is more difficult in the presence of anxiety: comorbidity lengthens the duration of treatment and decreases the response to interventions (49, 50). Our results may suggest one possible explanation: Specifically depressed youngsters with anxiety comorbidity use maladaptive ER responses to sadness even more frequently than their non comorbid peers, which is likely to worsen their dysphoria. Even though the comorbid group used maladaptive (total, behavioral, and social) responses more frequently, the effect sizes were low. This suggests that anxiety comorbidity is attributed only to a small proportion of variance in maladaptive ER strategies in youths with lifetime depression. One explanation could be that we examined the discriminating effect of anxiety comorbidity on ER domains within a depressed sample. Our results show a different style of maladaptive ER strategies in children and adolescents with lifetime anxiety and depression than in youngsters with only depression, namely, that cognitive maladaptive strategies are used to the same extent whereas social and behavioral maladaptive ER strategies are used more often in the comorbid group.

We found in our clinical sample that the presence or absence of anxiety comorbidity in patients with depression histories made no significant difference to these two groups’ use of adaptive ER responses. Our results are in line with those of Aldao et al. (12), whose meta-analysis found that adaptive ER strategies were less associated with psychopathology, including depression or anxiety, than were maladaptive ones. Therefore, patients with comorbidity may not derive benefits from expanding their repertoires of adaptive ER responses.

Our study can also serve to augment existing data on the possible role of dysfunctional ER in internalizing psychopathology. In our study of youths diagnosed with lifetime comorbid depression and anxiety, we examined not only their cognitive strategies but also their coping strategies in the social and behavioral domains. When comparing ER response clusters, we found that youths with comorbid anxiety and depression were more likely to use maladaptive social and behavioral/physical responses than were their depressed peers without anxiety. Social withdrawal (“hide from people”) within the Social domain can be considered as social avoidant strategy consistent with the nature of anxiety. As reported by Schafer et al. (11), avoidant behavior was associated with depressive and anxiety symptoms. While avoiding social situations to cope with sadness might reduce negative emotion in the short term, however, it also prevents children from using social support to attenuate sadness. Self-harm or autoaggression (e.g., “hit myself”) within the behavioral/physical domain can be interpreted as negative somatic sensitivity response. The utilization of these negative, emotion-driven somatic, sensory-focused responses may indicate a lack of more sophisticated regulatory strategies, which can be potentially harmful. It is also possible that anxiety, as one of the earliest forms of childhood psychopathology, might itself predispose individuals to more somatically based and sensory-oriented ER responses. This might be the subject of further research.

Finally, these findings suggest that patients with comorbid depression and anxiety may benefit from psychotherapeutic methods that aim to replace maladaptive ER responses with adaptive ones (51, 52, 53). One effective psychotherapy is dialectical behavioral therapy (DBT), which aims to improve ER by teaching several ER skills (e.g., emotion identification, increasing positive emotional events, taking opposite action, and distress tolerance techniques) (54). Promising results have been reported regarding the development (55, 56, 57) and effectiveness of transdiagnostic-behavioral therapy for children and adolescents with comorbid depression and anxiety (symptoms or disorder) (58). In the light of our results, the module of modifying maladaptive emotion-driven behaviors of transdiagnostic behavioral therapy could be especially beneficial for depressed children and adolescents with anxiety comorbidity (59).

### Limitations

Limitations of this research should also be mentioned. Since our study was cross-sectional, it was not possible to examine the causal relationship among the internalizing disorders and ER. We did not have enough subjects in current MDD and anxiety episodes in order to stratify our groups according to present or past diagnoses.

## Data Availability Statement

The datasets generated for this study are available on request to the corresponding author.

## Ethics Statement

This study was carried out in accordance with the recommendations of Federalwide Assurance for the Protection of Human Subjects with written informed consent from all subjects. All subjects gave written informed consent in accordance with the Declaration of Helsinki. The protocol was approved by the Institutional Review Board (IRB number: IRB00003344) and Medical Research Council (ETT TUKEB, nr: 444-0/2010-1018eku).

## Author Contributions

MK, KK, ÁV, EK, and IB conceptualized and received funding for the study. MK, KK, ÁV, EK, IB, EL, LB, IY, and JR planned and executed the study protocol. RD, KK, ÁV, EK, and IB contributed to data analysis and writing of the manuscript. RD, MK, KK, ÁV, EK, and IB assisted with the analysis of the data and contributed to the final draft of the manuscript.

## Funding

This study was supported by NIH Grant MH084938-01 and by Hungarian Scientific Research Fund Grant NN85285.

## Conflict of Interest

The authors declare that the research was conducted in the absence of any commercial or financial relationship that could be construed as a potential conflict of interest.
